# Eco-friendly impact of a novel green *Melilotus officinalis* extract as a sustainable inhibitor to reduce acid corrosion of copper

**DOI:** 10.1039/d4ra05391e

**Published:** 2024-11-21

**Authors:** Abd El-Aziz S. Fouda, Salah M. Rashwan, Medhat M. Kamel, Mohamed Atef, Ahmed El-Hossiany

**Affiliations:** a Department of Chemistry, Faculty of Science, Mansoura University Mansoura-35516 Egypt asfouda@mans.edu.eg; b Department of Chemistry, Faculty of Science, Suez Canal University Egypt; c Delta Fertilizers Company in Talkha Egypt

## Abstract

In this study, we deployed green *Melilotus officinalis* extract (MOE) as a corrosion inhibitor for copper. The anticorrosion properties of MOE for Cu in 1 M HNO_3_ were investigated by various experimental and numerical techniques, including potentiodynamic polarization (PDP), electrochemical impedance spectroscopy (EIS), and a weight loss (WL) method at different temperatures. Additionally, scanning electron microscopy (SEM) coupled with energy-dispersive X-ray analysis (EDX) and atomic force microscopy (AFM) were utilized to examine the surface morphology of Cu with and without the extract. By contrasting the inhibition effectiveness with and without the extract, the inhibition efficiency (% IE) was observed. The WL method revealed that 300 ppm of the extract had an IE of 93.8% for Cu immersed in one molar HNO_3_ solution. The MOE was classified as a mixed type according to the PDP study since it delayed both cathodic and anodic processes, with a cathodic predominance. At 25–45 °C, MOE's free adsorption energies were 23.1–21.5 kJ mol^−1^, showing that mixed-type adsorption occurred on the Cu surface. Additionally, the Langmuir adsorption isotherm and the adsorption data from the WL tests of MOE showed a good match. The extract could adsorb spontaneously onto the metal surface, according to the thermodynamic conditions. Analysis of the corrosion product using different techniques revealed that a protective layer had formed on the metal's surface. Hence, MOE had a good corrosive inhibitive effect on Cu in HNO_3_ solution. It turned out that all the methods used gave consistent results.

## Abstract 2

In this study, we deployed green *Melilotus officinalis* extract (MOE) as a corrosion inhibitor for copper.

## Graphical abstract

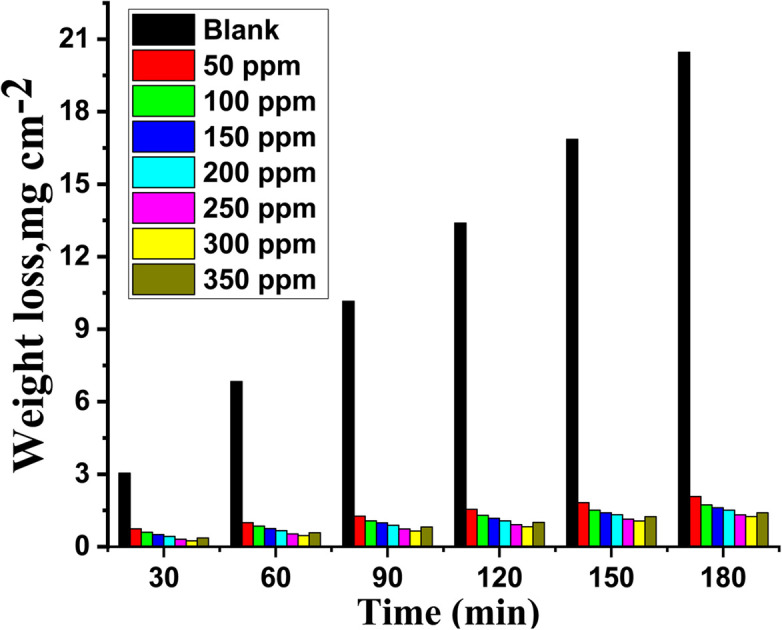

## 1. Introduction

Copper is one of the most commonly utilized metals for both industrial and residential applications, due to its many advantages, including its low cost, strong mechanical workability, and excellent electrical conductivity. However, copper can readily corrode due to a variety of environmental factors. The most common corrosive solution for copper is nitric acid.^1^ Corrosion is an electrochemical process involving anodic and cathodic reactions on the surface of metals.^2^ Any alteration in the physical components of metals, due to physicochemical contact with their environment, that results in deterioration of the metal's character is called corrosion.^3^ Corrosion is an expensive, as well as harmful issue. Every year, billions of dollars are spent on repairing corroded buildings, equipment, and parts as early failure could lead to the loss of human life and security damage. By adding chemical compounds to a corroding material, the surface metal corrosion can be controlled and minimized. The most effective method for dealing with corrosion of metals is using inhibitors. A corrosion inhibitor is a substance that, when added in small amounts to the corrosive solution, lowers the corrosion rate of the metal. In recent studies, not only have new inhibitors with high inhibition efficiency been developed, but also with other characteristics, such as low-cost, low-toxicity, and readiness for production processes.^4^ The use of inhibitors is the most practical method for defending against the successive dissolution of a metal by corrosion. The use of O-, S-, and N-containing organic compounds to minimize metal corrosion has been studied.^5^ The presence of heteroatoms in the inhibitor's chemical structure plays a significant role in the action of corrosion inhibition. Other factors have a significant influence on the efficiency of inhibition too, such as the molecular weight, aromatic rings, and inhibited metal load.^6^ Several papers were found in the literature related to the use of plant extracts as corrosion inhibitors for metals. For instance, the use of a pectin eco-friendly natural polymer as a corrosion inhibitor on mild steel in hydrochloric media demonstrated maximum inhibition efficiencies of 91.5% and 93.9% obtained using 1 g L^−1^ of both acid- and enzyme-extracted pectin, respectively.^7^ The extracts from *Aloe vera* leaves were studied in 1 M H_2_SO_4_ solution. The results indicated that the plant extract acts as a mixed-type inhibitor for mild steel with an IE% of 98% at a 30% v/v concentration.^8^ An extract from *Ricinus* was studied to reduce the mild steel corrosion in aqueous solution. The extract showed an IE% of 84% at a 300 ppm concentration.^9^ The effect of *Ficus carica* leaf extract on steel anticorrosion in petroleum solution was investigated,^10^ and the results indicated that the extract had an IE% of 70–80% at 298 K. The influence of *Citrus aurantium* leaves extract on mild steel corrosion in 1 M H_2_SO_4_ was studied by K. H. Hassan *et al.*^11^ Using the WL method, a maximum inhibition efficiency of 89% was obtained at 313 K with 10 mL L^−1^ of the leaves. Mango (*Mangifera indica*) extract was used as an inhibitor of the corrosion phenomenon affecting mild steel in 1 M H_2_SO_4_ in various methods.^12^ In this situation, the measurements were carried out at temperatures of 303–333 K. The results showed that the best inhibition efficiency of 74% was obtained at an inhibitor concentration of 0.97 g L^−1^ and temperature of 303 K. R. T. Loto^13^ investigated the inhibitory action of a combined admixture of *Rosmarinus officinalis* and zinc oxide on low carbon steel in 1 M HCl and H_2_SO_4_ solutions. Maximum inhibition efficiencies of 93.26% in HCl and 87.7% in H_2_SO_4_ acid solutions were observed. F. Bouhlal *et al.*^14^ analyzed coffee grounds extract as an inhibitor of the corrosion of C38 steel in 1 M hydrochloric acid medium. A high inhibition efficiency of 97.4% was achieved with 2 g L^−1^. H. Lahbib *et al.*^15^ used various methods to examine the inhibiting effect of dwarf palm and *Cynara cardunculus* leaves extract for St37 steel in 15% H_2_SO_4_ solution. High inhibition efficiencies of 76.13% and 69.35% were obtained with 30 ppm of dwarf palm and *Cynara cardunculus* leaves extracts, respectively. On the other hand, several authors (Table 1) have used plants extracts as inhibitors for copper to lessen metal corrosion in a variety of common industrial solutions.^16–30^

**Table 1:** Lists of plant extracts used as Cu corrosion inhibitors

Inhibitor (extract)	Medium	% IE	Ref.
*Moringa oleifera*	1 M H_3_PO_4_ + HNO_3_	73.5 at 300 ppm	16
*Idesia polycarpa* maxim fruits	0.5 M H_2_SO_4_	75–86	17
Cannabis plant	0.5 M H_2_SO_4_	91 at 25 ppm	18
Cinnamon essential oil	3 wt% NaCl	89.0	18
*Ephedra sarcocarpa* (ES)	2 M HCl	95 at 0.5 g L^−1^	19
Acid extract of *Azadirachta indica* seed	1–3 N HNO_3_	95 at 1% extract	20
Mangrove tannin	0.5 M HCl	82 at 3 g L^−1^	21
Caffeine isolated from black tea	0.5 NaCl	92	22
*Calligonum comosum*	2 M HCl	80.06% in 0.8 g L^−1^	23
Alcoholic extract of *Mimusops elengi* leaves	Natural sea water	86.84	24
*Alhagi maurorum* plant	0.5 M H_2_SO_4_	90	25
Olive leaf extract	0.5 M NaCl	90	26
*Capparis spinosa* L	Strong acid media	82.7 at 440 ppm	27
Ziziphus lotus	Sea water	93 at 5 g L^−1^	28
Berry leaves	2 M HNO_3_	90.1 at 300 ppm	29
Olive leaf	0.5 M NaCl	95	30
*Melilotus officinalis* (MOE)	1 M HNO_3_	93.8 at 300 ppm	Our results

tNevertheless, there are no further reports of MOE being used as an inhibitor. Therefore, the goal of this work was to estimate the inhibitory action of MOE against the corrosion of Cu in nitric acid solution using a WL method, and electrochemical techniques (potentiodynamic polarization (PDP) and electrochemical impedance spectroscopy (EIS)). These measurements were supplemented by SEM, EDX, AFM, and FTIR.

## 2. Experimental section

First, 1 M nitric acid (HNO_3_) was prepared in the laboratory by diluting an appropriate volume of analytical grade 69% HNO_3_ with bi-distilled water. For the WL tests, the composition of the Cu samples was: Sn (0.001), Fe (0.01), Bi (0.005), Pb (0.002), and Cu (rest) as a percentage by weight. Prior to every experiment, the Cu samples were washed with acetone and double-distilled water, dried, and ground using SiC sheets graded 600–1200. A Cu plate with the dimensions of 2 cm × 2 cm × 0.1 cm was used for the samples. For the electrochemical tests, the Cu sheet was welded with Cu wire for electrical connection and inserted into a Teflon tube and fixed with an adhesive. The area exposed to the solution was 1 cm^2^. The samples underwent treatment as before.

### 2.1 *Melilotus officinalis* (MOE) procedure

The fresh MOE parts were first prepared by dehydration, followed by milling to a powder. Then, 500 g of the powder was immersed in methanol, and boiled at 100 °C, and then left to stand at room temperature for about 72 h. After that, the MOE was filtrated and placed in the air to dry. In order to attain a 1000 ppm stock solution of the extract, 1 g of the dried MOE was dissolved in 1 L double-distilled water. In addition to the control, different dosages (from 50 to 300 ppm) were prepared from the stock solution and their anticorrosion effects were tested. The main components of MOE, comprising two new *p*-hydroxybenzoic acid glycosides, namely *p*-hydroxybenzoic acid-4-*O*-α-d-manopyranosyl-(1 → 3)-α-l-rhamnopyranoside and 4-*O*-α-L-rhamnopyran-osyl-(1 → 6)-α-d-manopyranosyl-(1 → 3)-α-l-rhamnopyranoside, and seven known compounds as acid components, flavonoids, a coumarin, and an alkaloid, were isolated from the 70% ethanol aqueous extract of the aerial parts of *Melilotus officinalis*.^31^

### 2.2 WL measurements

The American Society for Testing and Materials (ASTM) provides standardized procedures for conducting various types of corrosion tests, ensuring consistency and reliability in results.^32^ After being ground, cleaned, dried, and weighed, the Cu samples were suspended in 100 mL of 1 M HNO_3_ solution with and without different MOE dosages (50–350 ppm) for 60–360 min at different temperatures (25–45 °C) for investigation. After the designated exposure period, the Cu samples were removed, cleaned carefully with double-distilled water to eliminate the corrosion products, dried with filter papers, and weighed precisely according to the standard method. The loss in weight was estimated by the difference in weight of Cu samples before and after the exposure. Every measurement was performed in triplicate^33^ for assessing the reproducibility. The assessed WL can be used to determine the Cu surface area that is covered (*θ*) and the effectiveness of inhibition (% IE) by the following equation:1
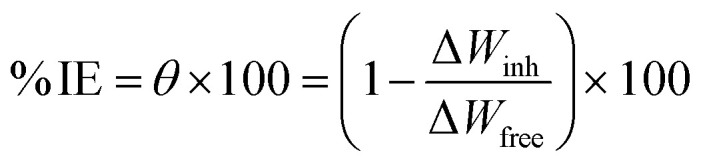
where the WL (mg) with and without MOE are represented by Δ*W*_inh_ and Δ*W*_free_, respectively. Following are the formulas for calculating the rate of the corrosion process (*k*_corr_):2
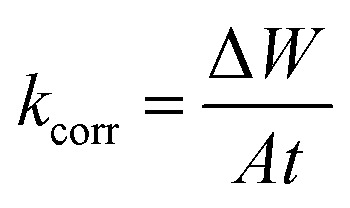
where *t* stands for the immersion period in minutes and *A* stands for the sample area in cm^2^. All the experiments were conducted in stagnant aerated solutions. The standard deviation values among parallel triplicate experiments were found to be smaller than 5%, indicating good reproducibility. All the experiments were carried out in stagnant aerated solutions.

### 2.3 Electrochemical procedures

A common glass cell was assembled with three electrodes was used for the electrochemical processes. The electrodes comprised a working electrode made of Cu sheet welded with Cu wire for electrical connection and inserted into a Teflon tube and fixed with an adhesive, and the area exposed to the solution was ∼1 cm^2^; a reference electrode made of SCE; and an auxiliary electrode made of Pt foil. The Cu electrode was immersed for 30 min in the solution prior to each electrochemical measurement so that the open circuit potential could reach a stable state.^34^ To record and retain data, the electrochemical procedures were done utilizing a potentiostat/galvanostat ZRA analyzer (PCI4/750, Gamry Instruments, Warminster PA, USA) and the analysis of the data from tests was performed using E-Chem Analyst 5.5 software (Gamry E-Chem Analyst Version 5.5 software 7.8.5.8567). An ultra-circulating thermostat was used to conduct each experiment at 25 °C without deaerating the solutions. Each experiment was conducted with a fresh Cu electrode and electrolyte.

#### 2.3.1 PDP measurements

The polarization test was performed by adjusting the potential ±250 mV *versus* OCP with a 0.2 mV s^−1^ sweep rate. The corrosion current density (*i*_corr_) and corrosion potential (*E*_corr_) were determined from Tafel plots without and with varying MOE doses. The following equation was used to get the % IE of MOE based on i_corr_ and the Cu surface coverage (*θ*):3
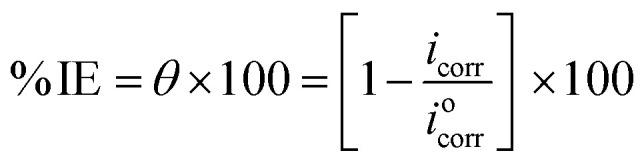
where, *i*^o^_corr_ and *i*_corr_, respectively, reflect the corrosion current's density without and with varying MOE doses.

#### 2.3.2 EIS measurements

The EIS test was accomplished with an alternating current amplitude of 10 mV in the frequency region of 10^5^–10^−1^ Hz. The % IE was derived from *θ* obtained from the EIS measurements using eqn (4):4
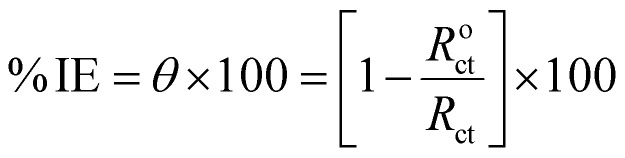
where *R*^o^_ct_ and *R*_ct_ stand for, respectively, the resistance to charge transfer without and with varying Sumac extract doses.

### 2.4 Surface analysis

The Cu surface was analyzed using SEM, EDX, AFM, and FTIR before and after 3 h of immersion in a corrosive acidic environment without and with 300 ppm (highest dose) MOE. Scanning electron microscopy (SEM) and energy-dispersive X-ray analysis (EDX) were performed on a JEOL device (JSM IT – 100, Japan). Atomic force microscopy (AFM) was also applied as it is an effective method for studying the surface morphology of Cu alloys at the nano or micro scale, and the data were analyzed using Nanosurf C3000 software, version 3.5.0.31. Fourier-transform infrared spectroscopy (FTIR) measurements were carried out using the total reflectance (ATR) mode and an iS 10 FTIR spectrometer from Thermo Fisher Scientific (USA).^35^

## 3. Results and discussion

### 3.1 WL method

**Fig. 1:**
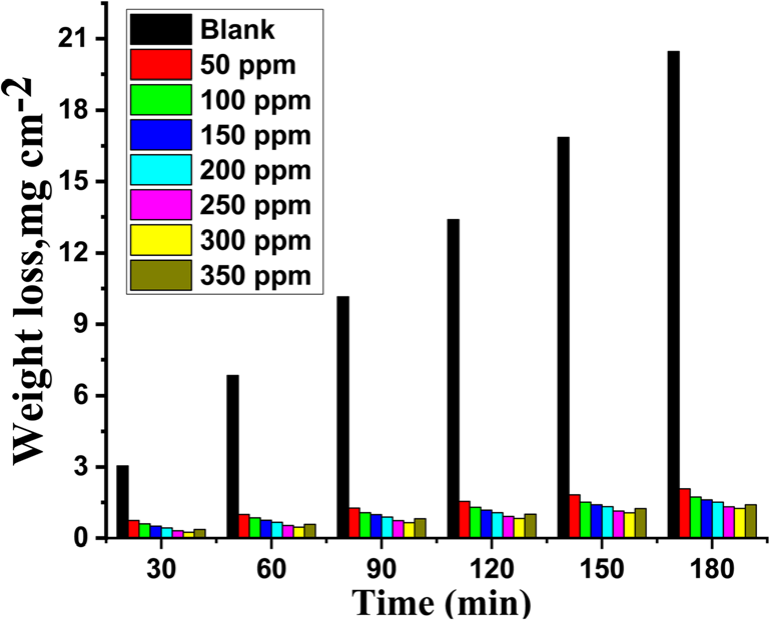
WL *versus* time bar chart for the corrosion of Cu in 1 M HNO_3_ in the presence and absence of varying doses of MOE at 25 °C.

The effect of adding varying amounts (50–350 ppm) of MOE on Cu corrosion in a 1 M HNO_3_ solution was evaluated using the WL method. The relationship between the impacts of various doses of MOE on Cu given by the WL *vs.* time at 25 °C is depicted in Fig. 1. It was evident that the WL of Cu in the presence of the extract was significantly lower than that obtained in the blank solution. Additionally, Fig. 1 shows that the rate of Cu corrosion in nitric acid increased with the increase in contact time, but was reduced with the increase in MOE dosage, showing that the extract molecular layer adsorbed on the Cu surface caused a drop in WL. This layer protects the metal's corrosive sites and keeps the metal from corroding in the corrosive environment.^36,37^ This method has proven to be the most accurate method for determining the inhibition efficiency (% IE) and corrosion rate (*k*_corr_). According to the data in Table 2, increasing the dose of MOE up to 300 ppm caused the corrosion rate to decrease and IE to increase. The addition of a concentration of 350 ppm led to a lower inhibition efficiency than at 300 ppm. Because of the physical adsorption process, the inhibition efficiency decreased to 92.5%, as indicated in Table 2. The solution became saturated and the inhibition efficiency decreased when the inhibitor concentration surpassed 300 ppm, which was this considered the optimum concentration. Additionally, there is a chance that the molecular extract in the solution and the extract affixed to the metal surface could interact strongly.^38,39^ The coating formed from the extract may be released into the solution as a result of this interaction. Since the amount calculated is directly proportional to the level of resolution, the WL test is typically chosen. In the solutions with and without extract, the linear fluctuation of WL over time showed that there were no insoluble surface layers caused by corrosion, whereby the inhibitor MOE was first adsorbed on the surface of the Cu alloy and subsequently reduced.

**Table 2:** *k*_corr_, *θ*, and % IE for the varying doses of MOE at 25 °C

Conc., ppm	*k*_corr_ mg cm^−1^ min^−1^	*θ*	% IE
Blank	0.11159	—	—
50	0.01286	0.885	88.5
100	0.01084	0.903	90.3
150	0.00982	0.912	91.2
200	0.0087	0.922	92.2
250	0.00757	0.932	93.2
300	0.00690	0.938	93.8
350	0.00837	0.925	92.5

### 3.2 Temperature effect

Through the use of WL tests, the impact of different temperatures (25 °C, 30 °C, 35 °C, 40 °C, and 45 °C) on the corrosion of Cu in 1 M HNO_3_ in the presence and absence of different doses of MOE extract was investigated. Table 3 displays the estimates of the CR and % IE derived from the WL tests for different doses of the extract in 1 M HNO_3_ solution at different temperatures. The findings show that an increase in the extract dose led to a decrease in CR value and an increase in % IE. This is a result of the extract's increasing degree of adsorption and subsequent covering of the Cu surface as the extract dosage was increased.^40^ The decrease in % IE with increasing the temperature shows that the extract may be adsorbed physically on the Cu surface.

**Table 3:** Results of the WL tests (*k*_corr_, in mg cm^−2^ min^−1^, *θ*, and % IE) for Cu in the presence and absence of MOE after 120 min at 30–45 °C

Con., ppm	30 °C		35 °C		40 °C		45 °C	
	*k*_corr_	% IE	*k*_corr_	% IE	*k*_corr_	% IE	*k*_corr_	% IE
Blank	0.1473	—	0.2188	—	0.2848	—	0.3275	—
50	0.0202	86.4	0.0343	84.3	0.0527	81.5	0.0690	78.9
100	0.0188	87.3	0.0316	85.5	0.0473	83.4	0.0634	80.7
150	0.0171	88.4	0.0286	86.9	0.0436	84.7	0.0611	81.4
200	0.0154	89.6	0.0253	88.5	0.0410	85.6	0.0562	82.9
250	0.0140	90.5	0.0231	89.5	0.0367	87.1	0.0502	84.7
300	0.0119	91.9	0.0202	90.8	0.0308	89.2	0.0427	87.0

**Fig. 2:**
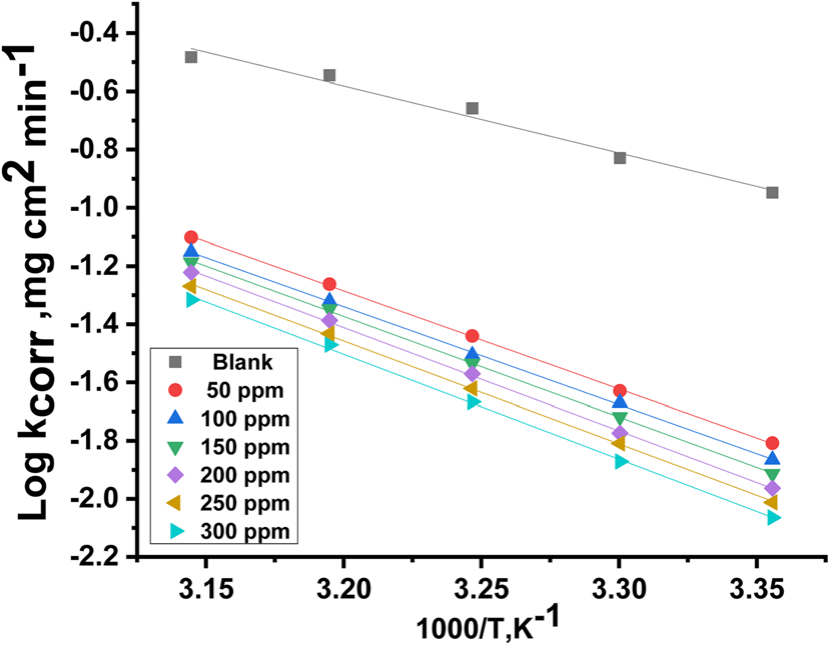
Plot of log *k*_corr_*versus* 1/*T* for Cu in the presence and absence of varying doses of MOE.

The estimation of 
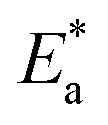
 for the corrosion of Cu in uninhibited and inhibited acid corrosive media was computed by employing the Arrhenius equation:^41^5
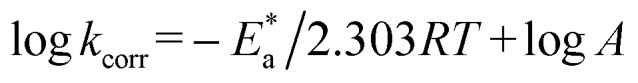
where *A* is the pre-exponential Arrhenius constant. Straight lines were achieved by drawing log *k*_corr_*versus* 1/*T* without MOE and at different doses of MOE (Fig. 2) with a slope of 
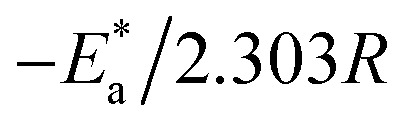
. The entropy (ΔS*) and enthalpy (ΔH*) of activation of Cu corrosion in a corrosive acidic environment without and with different doses of MOE were calculated using eqn (6):^42^

**Fig. 3:**
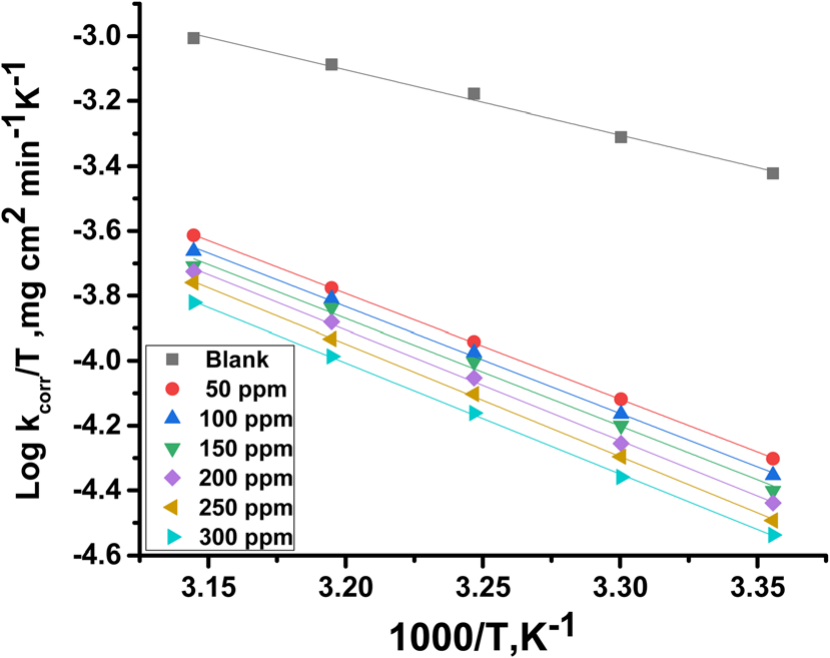
Plot of log *k*_corr_/*T* against 1/*T* for Cu in the presence and absence or varying doses of MOE.

6 *k*_corr_ = *RT*/*Nh* exp^(ΔS*/*R*)^ exp ^(−ΔH*/*RT*)^

where *h* represents Planck's constant. Plotting log *k*_corr_/*T vs.* 1/*T* with and without an adjusted dose of MOE yielded straight lines (Fig. 3). Table 4 displays the highest values of 
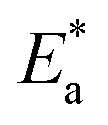
 and ΔH* when MOE was added, suggesting that the physical adsorption of MOE took place on the Cu surface. Positive values for ΔH* indicate an endothermic adsorption of MOE molecules on the Cu. Large and negative values of ΔS* in the uninhibited and inhibited system imply that the activation complex in the rate-determining steps represented an association rather than dissociation step, meaning that a decrease in disordering took place on going from the reactants to the activated complex.^43,44^

**Table 4:** 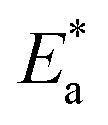
, ΔH*, and ΔS* for Cu in the presence and absence of varying doses of MOE

C_inh_, ppm	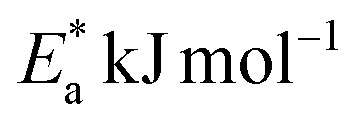	ΔH* kJ mol^−1^	−ΔS* J mol^−1^ K^−1^
Blank	46.9	44.6	120
50	65.9	63.4	98
100	67.7	65.3	94
150	69.2	66.6	91
200	69.4	66.3	90
250	70.0	67.4	88
300	70.7	68.6	88

### 3.3 Adsorption consideration

**Fig. 4:**
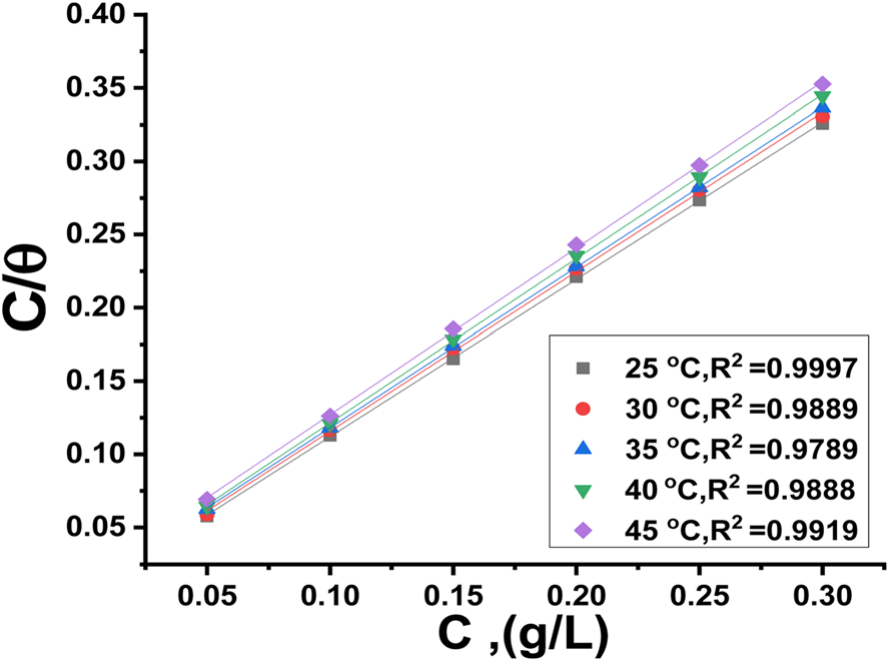
Langmuir isotherm model for MOE for the liquefaction of Cu in 1 M HNO_3_.

To identify the corrosion mechanism, numerous adsorption isotherms have been used. Several adsorption isotherm equations, including the Langmuir, Freundlich, Temkin, and Frumkin, were solved using the experimental data derived using the WL approach. The isotherm that fitted the experimental data the best was selected using the correlation coefficient (*R*^2^). The results were consistent with Langmuir's isotherm (Fig. 4) for adsorption (eqn (7)), which uses the subsequent equation to relate the metal surface coverage (*θ*) to the additive dose (*C*_inh_):7
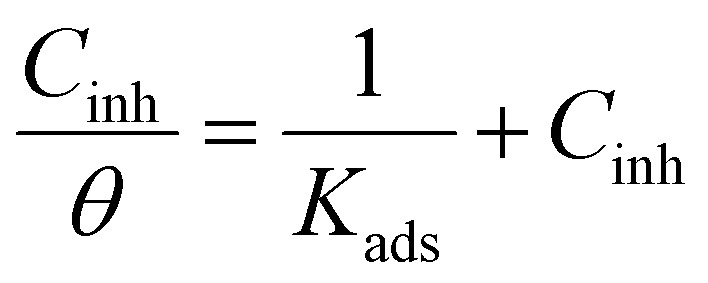
Within the given context, the dose of the inhibitor (measured in ppm) inside the bulk electrolyte is indicated as *C*, the surface coverage degree is *θ*, and the adsorption constant is *K*_ads_. Plotting *C*/*θ* against *C* revealed that the slope of the Langmuir adsorption isotherm was close to unity and that the result showed the intercept was 1/*K*_ads_. The slope's deviation from unity can be explained by the reciprocal repulsion or attraction between the adsorbed molecules of the extract on the Cu surface.^45^ By utilizing eqn (7), the values of (*K*_ads_) were determined. The Langmuir adsorption isotherm was chosen as the best isotherm fitting the experimental data based on the high correlation coefficient (*R*^2^ > 0.9789). The adsorption constant, *K*_ads_, obtained from eqn (8) of the Langmuir adsorption isotherm, is specifically related to the free energy of adsorption, 
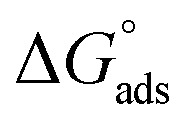
, by the relation:8
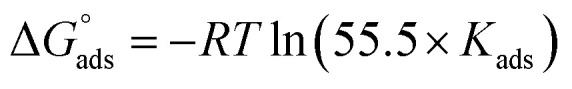
where the numerical value 55.5 corresponds to the solution's water molarity, indicating the number of water particles per unit volume, measured in M^−1^ units. As is known, one crucial thermodynamic parameter for characterizing the adsorption process is 
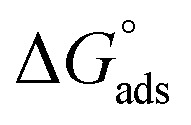
. Table 5 lists the adsorption parameters for the obtained MOE. The achieved negative values of 
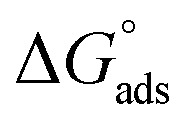
 decreased with increasing the temperature, indicating the greater stability of the adsorbed layer at low temperature, as shown by the data in the Table 5, indicating the spontaneous action of the MOE on the surface of the Cu. The 
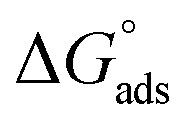
 value was less than 20 kJ mol^−1^, indicating physical adsorption, with values ranging from 20 to 40 kJ mol^−1^ indicating mixed adsorption (physical and chemical). Similar to the results obtained,^46^ the value of 
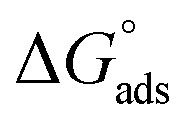
 in the current data was 23.1 kJ mol^−1^, showing a mixed type inhibitor with physisorption being more relevant.

**Table 5:** Langmuir parameters for the adsorption of MOE on the Cu surface at varied temperatures

Temp., K	*K*_ads_ M^−1^	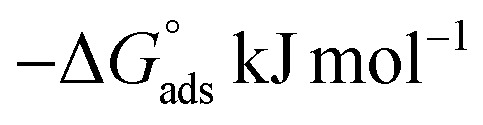	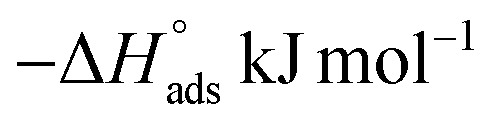	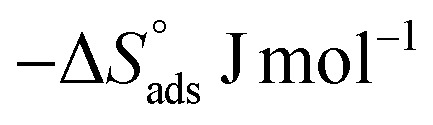
298	198.1	23.1	47.0	77.2
303	153.6	22.8		75.1
308	113.2	22.3		72.5
313	81.4	21.9		69.8
318	61.5	21.5		67.5

The enthalpy of adsorption (
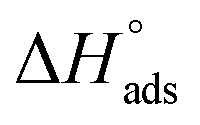
) was computed utilizing the following Vant Hoff equation:9
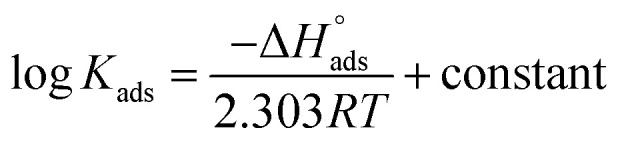


**Fig. 5:**
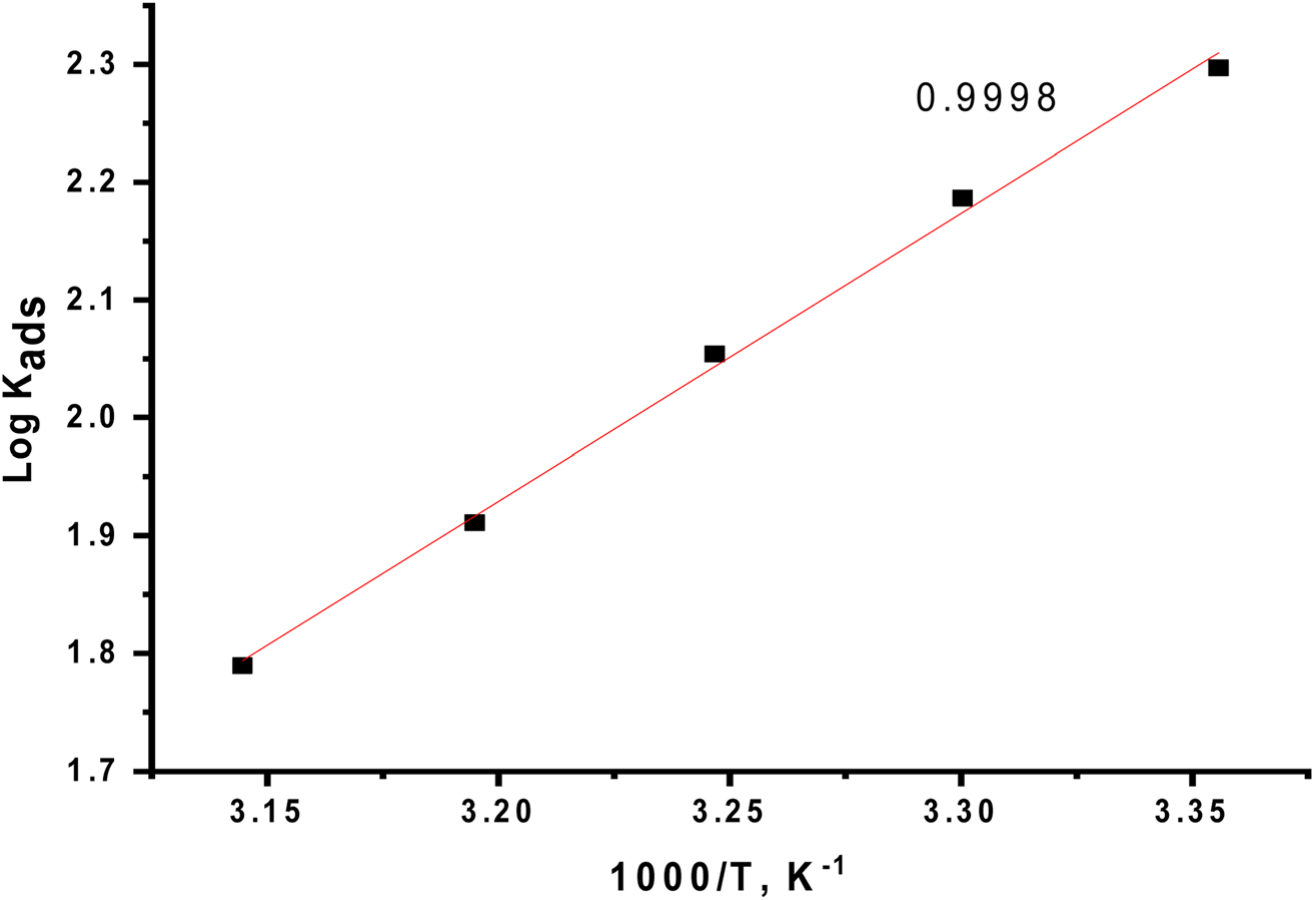
Effect of varying the temperature on the free energies of the Langmuir isotherm.

Plotting log *K*_ads_*versus* 1/*T* produced a straight line, as shown in Fig. 5. The adsorption entropy (
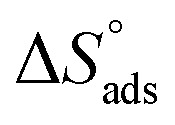
) can be calculated as follows:10
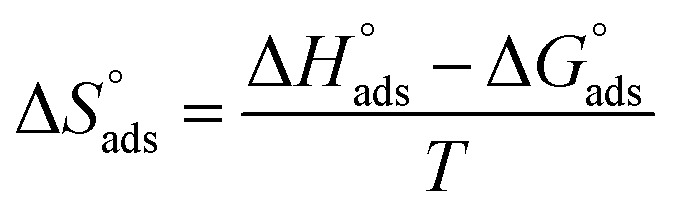


Table 5 displays the computed 
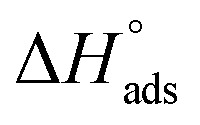
 and 
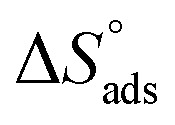
 values. The exothermal adsorption of MOE on the Cu surface was indicated by the negative sign of 
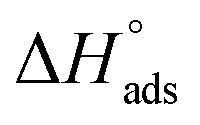
. Adsorption can be chemical or physical in an exothermic procedure. The negative sign of 
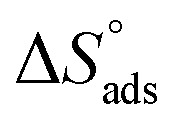
 indicated reduced turbulence upon transition from the reactant to the adsorbed metal. This supports the potency and adsorption power of MOE.

### 3.4 PDP tests

**Fig. 6:**
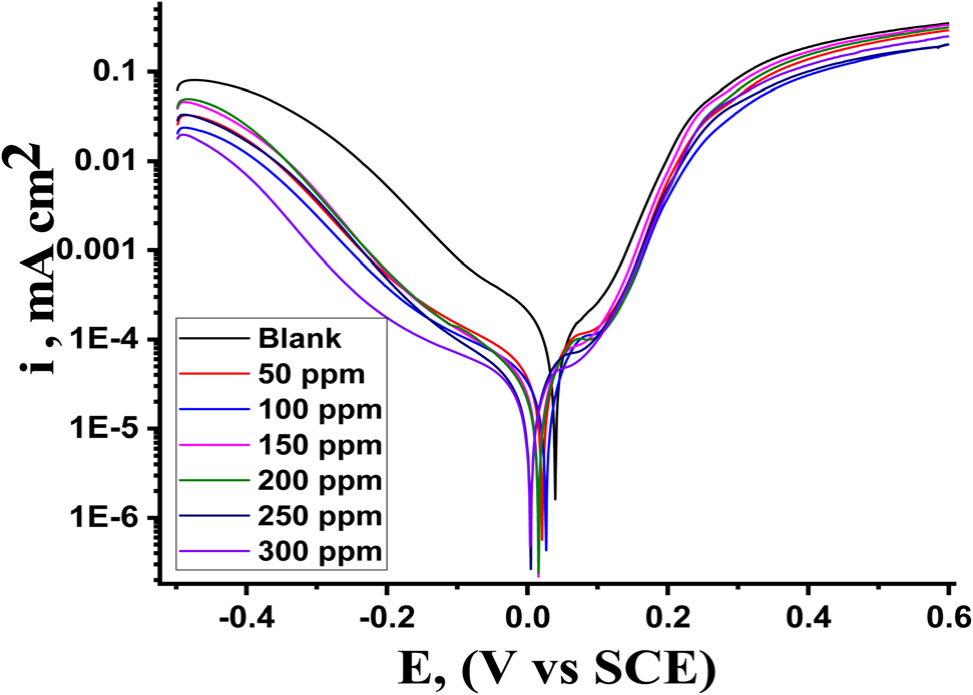
Illustration of the PDP curves showing the dissolving behavior of Cu in the presence and absence of MOE extract at 25 °C.

PDP is an analytical technique that is essential to the study of corrosion inhibition because it yields important information regarding the tendency of a material toward passivation or pitting, as well as its rate of corrosion. By contrasting the polarization curves produced with and without inhibitors, it also aids in estimating the efficacy of corrosion inhibitors. Polarization data were produced from the PDP measurement and utilized to examine the adsorption mechanism. Fig. 6 displays the PDP curves for Cu corrosion in 1 M HNO_3_ with and without varying doses of MOE at 25 °C, showing that the anodic and cathodic plots moved to lower values, but largely on cathodic curves, indicating that the effect of the extract on the cathodic current was more pronounced than its effect on the anodic current. MOE can therefore, be regarded more as a cathodic-type inhibitor. According to the Stern–Geary theory,^47^ a stronger corrosion resistance should also result from higher (*β*_a_) and (*β*_c_) values as depicted from Table 6, corresponding to lower i_corr_ values. According to Li *et al.*,^48^ an inhibitor must have a minimum change in *E*_corr_ of ±85 mV in order to be classified as either cathodic or anodic. The greatest displacement in this investigation was 28 mV, indicating that MOE was a mixed-type inhibitor. This finding implies that MOE was adsorbed by reaction at the Cu sites, which decreased the surface area available for the cathodic reactions and metal dissolution. The corrosion rate was computed using these parameters. The results of the electrochemical parameters at different MOE doses are listed in Table 6. The addition of 300 ppm produced the best inhibitory efficiency value of 91.1% (Table 6). This finding suggests that raising the dose to 300 ppm will effectively slow down the pace of Cu corrosion. The relationship between the rise in inhibition efficiency and the fall in current density was linear. The adsorption mechanism was examined using the PDP measurement findings. The anodic (*β*_a_) and cathodic (*β*_c_) Tafel slope values were essentially unchanged and parallel, demonstrating that the extract's addition had no effect on the mechanism. The *i*_corr_ values decreased as the extract dosages increased, suggesting that the percentage of IE rose as the extract dosage increased, demonstrating a respectable level of consistency with the results from alternative methods.

**Table 6:** Calculations of the corrosion parameters for Cu in 1.0 M HNO_3_ with varying inhibitor doses

Conc. ppm	*i*_corr._ μA cm^−2^	*E*_corr._, mV *vs.* SCE	*β*_a_, mV dec^−1^	−*β*_c_, mV dec^−1^	*k*_corr_, ×10^2^ mpy	*θ*	% IE
Blank	985	49	123	185	420	—	—
50	205	21	117	214	187	0.792	79.2
100	184	26	107	245	177	0.813	81.3
150	121	21	124	195	168	0.877	87.7
200	110	16	106	169	112	0.888	88.8
250	101	11	116	159	92	0.897	89.7
300	88	8	114	199	74	0.911	91.1

### 3.5 EIS test

**Fig. 7:**
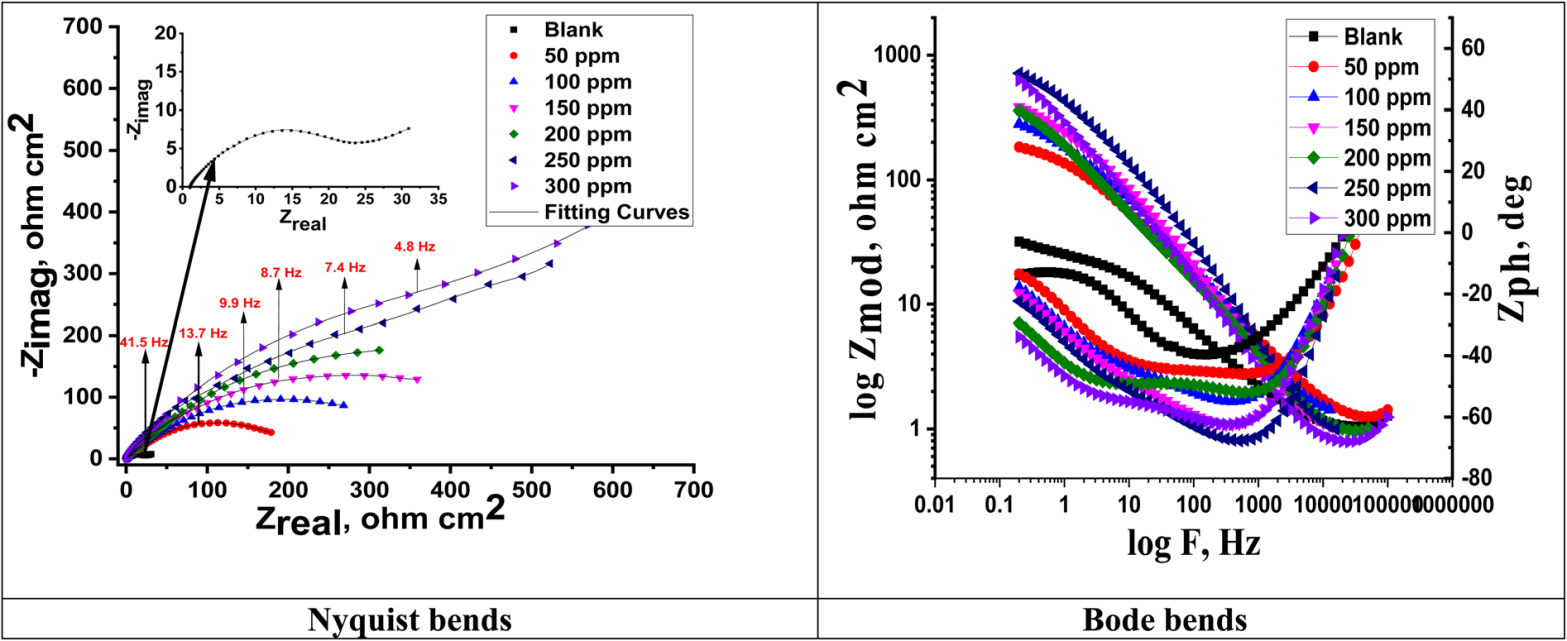
Nyquist and Bode curves for Cu liquefaction in 1 M HNO_3_ in the presence and absence of varying doses of MOE at 25 °C.

**Fig. 8:**
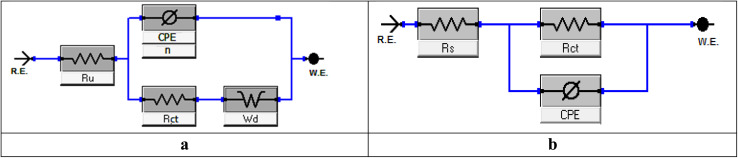
Equivalent circuit to fit the EIS data for Cu displaying a Warburg impedance in 1 M HNO_3_ (a). Equivalent circuit to fit the EIS data for Cu displaying one or more capacitive loops with MOE (b).

Fig. 8 shows the electrochemical impedance (EIS) data that were examined using the suggested equivalent circuit diagram for Cu immersed in the test solution with and without different extract dosages. This figure shows the attained Nyquist and Bode curves for Cu dissolution in 1 M nitric acid with and without varying doses of MOE. A semicircle loop was used to identify the Nyquist diagrams, and proved that a charge-transfer process was followed for Cu metal disintegration.^49^ Perfect semicircles were not produced by the Nyquist plot as predicted by the EIS theory. Both the surface inhomogeneities and the frequency dispersion are typically cited as the causes of the departure from the ideal semicircle.^50^Fig. 7 shows that the low-frequency area, where the impedance values grew in the presence of MOE compared to the lack of MOE, the radius of the circle rose as the dosage of MOE was increased, and therefore the charge-transfer resistance to the corrosion reactions increased. Because of the adsorption of MOE at the Cu/solution contact,^51^ a high resistance was produced. The Bode graphs (Fig. 7) demonstrated how the phase angle shift continuously increased, which was clearly correlated with the increase in MOE adsorbed on the Cu surface. They also illustrate how the overall impedance increased with increasing MOE (log *Z vs.* log *f*) and (log *f vs.* phase). Additionally, the Bode plots revealed a single peak component, suggesting that the one-time constant equivalent model with CPE provided the best fit for the EIS results. Furthermore, the Bode plots for the extract only showed one phase maximum associated with a single relaxation event, which was most likely caused by the charge-transfer process occurring at the metal/electrolyte interface. The matching circuits for the Cu and electrolyte are shown in Fig. 8a and b. Fig. 8a represent the equivalent circuit of the acid only and it consists of electrolyte resistance (*R*_u_), charge-transfer resistance (*R*_ct_), constant phase element (CPE), and *W*_d_ (the quadratic resistance, *W*_d_ = 88.69 × 10^−3^ Ω cm^2^). Fig. 8b represents the equivalent circuit for the corrosive solution containing MOE. It depicts a single charge-transfer reaction and is in good agreement with the outcomes of our experiments. To provide a more precise fit, the circuit incorporated a constant phase element (CPE) *in lieu* of a pure double-layer capacitor.^52^ CPE (*Y*_0_ and *n*) was determined by the next equation to estimate the interfacial capacitance *C*_dl_ data:

11 *C*_dl_ = *Y*_0_(*ω*_max_)^*n*−1^

where *Y*_0_ is the CPE magnitude, *n* is the variance CPE data (*i.e.*, −1 ≤ *n* ≤ 1), *ω* is the angular frequency (*ω*_max_ = 2π*f*_max_), and *f*_max_ is the maximum frequency. The parameters obtained by fitted Nyquist arcs are summarized in Table 7. Aggressive species access to the metal surface was prevented by the adsorption of the extract on the Cu surface, which blocked the active sites.^53^ As the dosage rose, the homogeneity parameter *n* increased as well, showing a decrease in surface roughness and heterogeneity.^54^ The impedance coefficients for Cu in 1 M nitric acid in the presence and absence of the varying doses of MOE were obtained. Table 7 shows there was a decrease in *C*_dl_ values and increase in *R*_ct_ values as the MOE dose increased, which could be explained by the decrease in the local dielectric constant and/or an increase in the thickness of the electrical double layer.^55^ This was due to the MOE molecules adsorbing on the Cu/interface with the solution and generating a protective coating on the Cu solution's interface.

**Table 7:** Impedance parameters for Cu after immersion in 1.0 M HNO_3_ in the presence of varying MOE doses at 25 °C

Conc., ppm	*Y*_o_, (μΩ^−1^ s^*n*^ cm^−2^)	*n*	*R*_ct_, Ω cm^2^	*C*_dl_, μF cm^−2^	*W*_d_ Ω cm^2^	*θ*	% IE	Goodness of fit, (*χ*^2^)
Blank	518	0.961	29	436	88.69 × 10^−3^	—	—	20.32 × 10^−3^
50	319	0.973	186	295	—	0.641	64.1	22.47 × 10^−3^
100	229	0.978	231	214	—	0.698	69.8	17.75 × 10^−3^
150	187	0.987	279	179	—	0.801	80.1	20.14 × 10^−3^
200	108	0.989	349	104	—	0.835	83.5	19.45 × 10^−3^
250	101	0.991	522	98	—	0.856	85.6	14.7 × 10^−3^
300	91	0.995	601	89	—	0.903	90.3	13.89 × 10^−3^

### 3.6 Surface analysis

#### 3.6.1 AFM studies

**Fig. 9:**
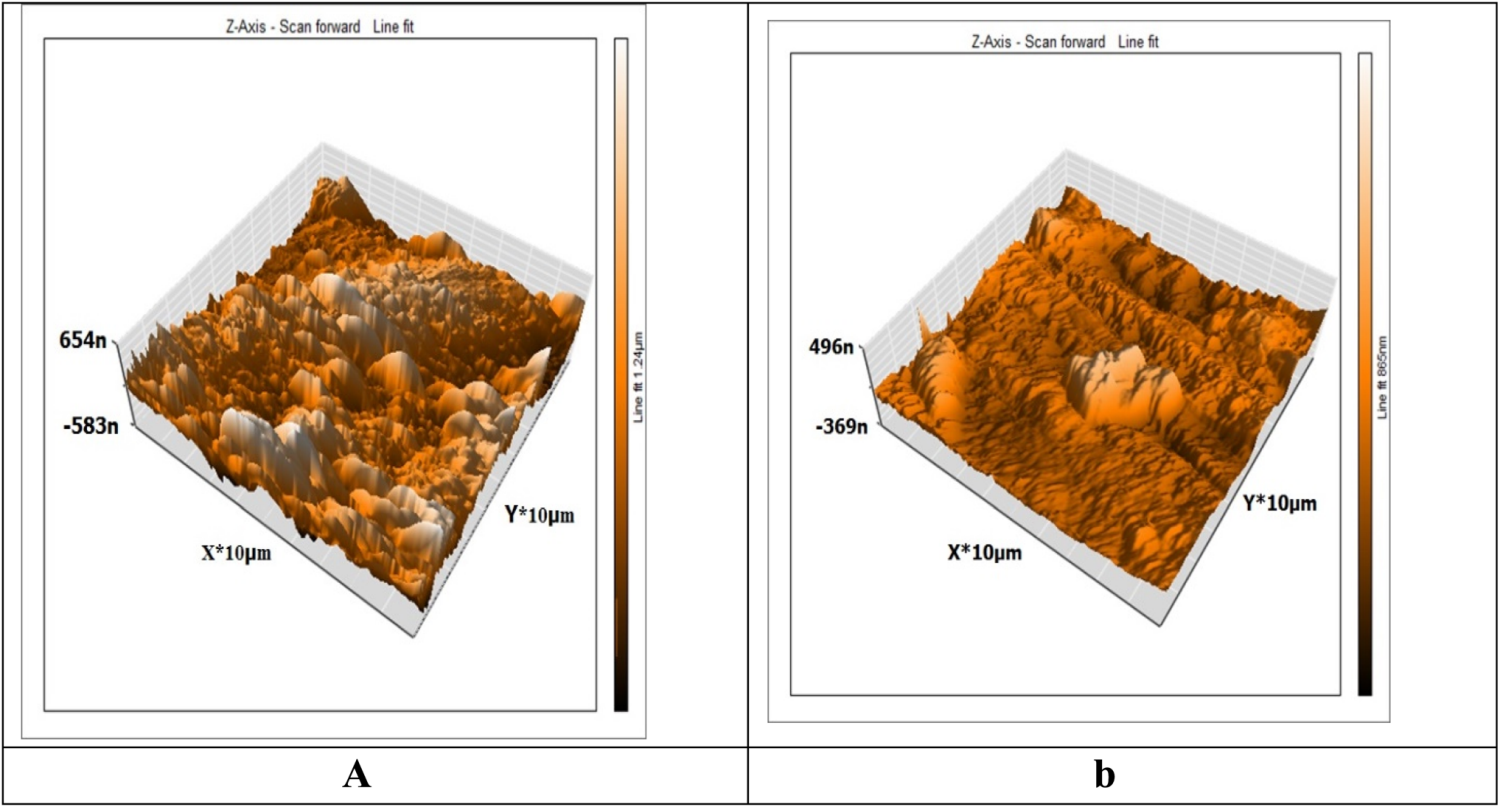
3D AFM images for Cu specimen in 1 M HNO_3_ only (a) and Cu specimen + 1 M HNO_3_ + 300 ppm of MOE after 3 h immersion (b).

An effective method for examining the surface topography at the nano-to-micro scale is AFM. The inhibitor influence on the formation and advancement of corrosion at the metal or GLSS/solution contact has emerged as a novel research avenue.^56,57^ AFN is increasingly being used as a recognized technique to examine the roughness of metals, alloys, and glasses.^58^ The surface morphology of the Cu was analyzed by AFM experiments after dipping in 1 M HNO_3_ in the presence of 300 ppm MOE after 3 h immersion and was determined in terms of the surface roughness. The average values of the “*S*_a_” roughness profile play a significant role in recognizing and reporting the effectiveness of a test inhibitor.^59^ Among other things, roughness plays a role in explaining the nature of the layer adsorbed on Cu. Fig. 9a shows the metal surface (white) was damaged by HNO_3_, which had a high roughness value (*S*_a_ = 277), while Fig. 9b shows the Cu surface with the presence of 300 ppm MOE, which was not so corroded and had a lower roughness value (*S*_a_ = 52).

#### 3.6.2 FTIR spectra

**Fig. 10:**
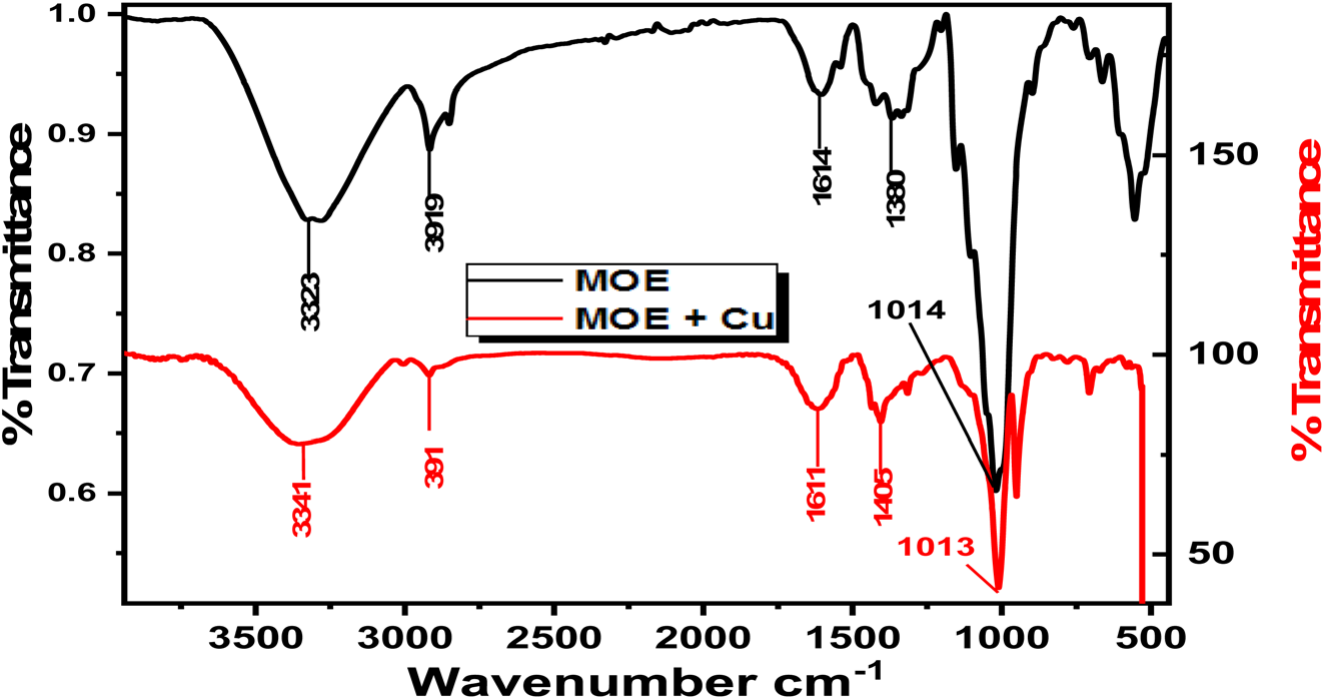
FTIR spectra for free MOE (a) and the metal with MOE (b).

It is commonly known that the FTIR spectrophotometer is an effective instrument for determining the kind of bonding, namely the functional group(s), present in organic molecules. FTIR can be used to determine the type of chemical components adsorbed on the surface by analyzing surface variations. The interaction between the extract molecules and the metal surface was determined by FTIR analysis.^60^ The FTIR spectra of an extract of MOE both before and after their adsorption on the Cu surface are shown in Fig. 10. The stretching vibration of the O–H stretch corresponded to the peak at 3343 cm^−1^. This peak was modified to 3355 cm^−1^ in the spectrum of the sample collected from the metal surface. It could be seen from Fig. 10 that there was a change in frequencies between the spectra of the MOE and the corrosion product after Cu was immersed, suggesting that the MOE interacted with the Cu surface through the functional groups of the extract.

#### 3.6.3 SEM studies

**Fig. 11:**
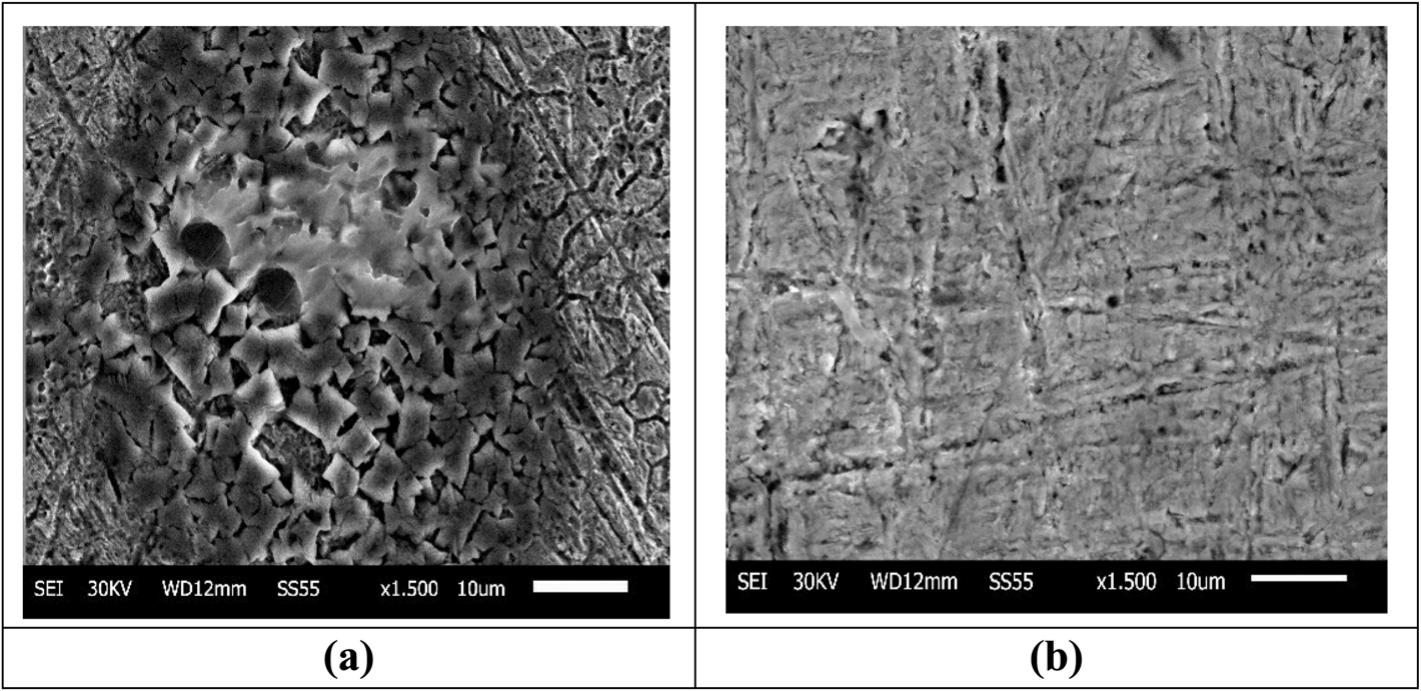
SEM micrographs of Cu after one day of immersion in 1 M HNO_3_ only (a), Cu after one day of immersion in 1 M HNO_3_ with 300 ppm MOE (b).

The morphology of the Cu samples before corrosion was smooth with scratches due to use of the emery paper. When the samples were immersed in 1 M HNO_3_, it resulted in the complete degradation of the microstructure of the Cu surface, leading to the formation of significant cracks, as shown in (Fig. 11a).^61^ On the other hand, when MOE was added, the extract molecules yielded a notable reduction in Cu damage^50^ and the surface became smooth, resulting in complete surface coverage,^62^ as demonstrated in Fig. 11b.

#### 3.6.4 EDX spectroscopy

**Fig. 12:**
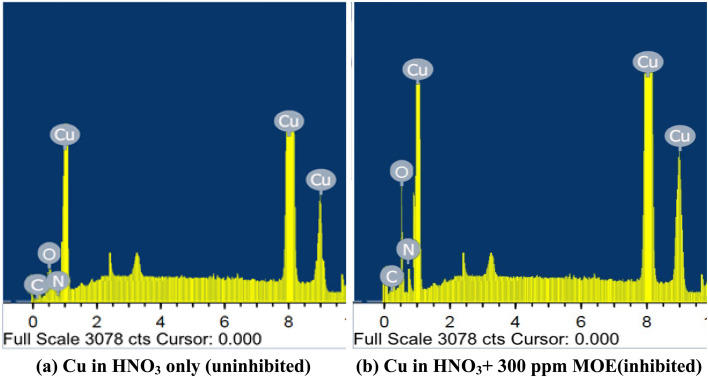
EDX analysis of Cu after exposure to 1 M HNO_3_ (a) and in the presence of MOE (300 ppm) after one day immersion.

The quantitative dose of the primary components found on the Cu surface could be determined from the EDX spectra.^63^Fig. 12 displays the EDX spectra for the Cu surface after one day of immersion in 1 M HNO_3_ without and with 300 ppm MOE. Table 8 lists the spectral elements' mass percentages. The spectrum of the Cu surface without MOE proved the existence of the elements O, C, and Cu. The inhibited surface's spectrum (Cu surface in HNO_3_ solution with MOE) showed that there were more O and N atoms present, indicating that MOE had been adsorbed on the Cu surface, generating a layer that shielded the Cu from HNO_3_ attack. The analysis results firmly back the usage of MOE in 1 M HNO_3_ as a Cu corrosion inhibitor.

**Table 8:** Cu surface components (mass%) prior to and after exposure to 1 M HNO_3_ for one day without and with the presence of MOE (300 ppm)

Mass percentages	O	C	Cu	N
Uninhibited Cu surface	18.18	5.79	58.21	6.18
Inhibited Cu surface	23.04	7.98	69.33	7.56

### 3.7 Mechanism of corrosion inhibition

**Fig. 13:**
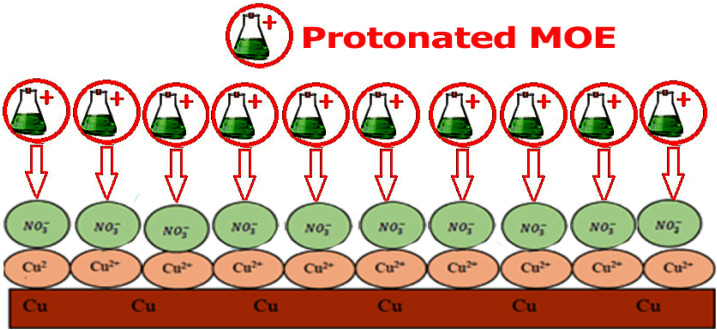
Diagram showing the adsorption behavior of the extract.

The adsorption process is influenced by various factors, including the interaction between Cu and the solution, the electrochemical potential, the chemical composition, and the surface properties of Cu. The adsorption phenomena are generally influenced by the charge on the metal surface, the kind of contact with the metal surface, and the chemical structure of the inhibitor. An adsorbent typically adheres to a surface by forming bonds or through chemical or physical adsorption. The extract adsorption on the Cu surface immersed in HNO_3_ is a part of the inhibition mechanism. MOE may be found at the metal-solution contact from four different types of adsorption.^64^ From the observations drawn from the various tests, corrosion hindrance of Cu in 1 M HNO_3_ solutions by MOE, as noted from the WL, PDP, and EIS tests, was found to rely on the dosage and the nature of the extract. Some molecules of MOE may be protonated in the acid medium. So, it is difficult for these protonated molecules to adsorb on the positive Cu surface.^65,66^ Nitrate ions get adsorbed first on the Cu surface, and so the Cu surface becomes negatively charged, and then the protonated molecules of MOE get adsorbed on the nitrate layer, as shown below, forming a physisorption mechanism. Fig. 13 shows the adsorption behavior of the extract.

## 4. Conclusions

This work evaluated the inhibitory action of MOE for Cu in HNO_3_ solution utilizing WL tests, and PDP and EIS techniques. The inhibition efficiency obtained from all the measurements showed fairly good agreement. The polarization experiments showed that the extract from MO functioned as a mixed-type inhibitor, delaying both cathodic and anodic processes without altering the corrosion mechanism. Impedance measurements demonstrated that the organic components of the MOE were adsorbed onto the corroding metal surface. It was discovered that the Langmuir adsorption isotherm was followed by the adsorption process from the acid. The values of the activation kinetic parameters derived from the experimental data supported the adsorption of MOE at various temperatures. The adsorbed protective film's AFM and FTIR morphology results on the surface of Cu verified MOE's high inhibitive effect.

## Data availability

The data that support the findings of this study are available on request from the corresponding author.

## Author contributions

Abd El-Aziz S. Fouda wrote the main manuscript text, Salah M. Rashwan supervision, Medhat M. Kamel and Mohamed Atef carried out the experimental part, Ahmed El-Hossiany, prepare the Tables and Figures. All the authors reviewed the manuscript.

## Conflicts of interest

There are no conflicts to declare.
